# Causal associations of thyroid function and dysfunction with overall, breast and thyroid cancer: A two-sample Mendelian randomization study

**DOI:** 10.1002/ijc.32988

**Published:** 2020-04-03

**Authors:** Shuai Yuan, Siddhartha Kar, Mathew Vithayathil, Paul Carter, Amy M. Mason, Stephen Burgess, Susanna C. Larsson

**Affiliations:** 1 Unit of Cardiovascular and Nutritional Epidemiology, Institute of Environmental Medicine, Karolinska Institutet, Stockholm, Sweden; 2 Department of Surgical Sciences, Uppsala University, Uppsala, Sweden; 3 Department of Public Health and Primary Care, University of Cambridge, Cambridge, United Kingdom; 4 MRC Integrative Epidemiology Unit, Bristol Medical School, University of Bristol, Bristol, United Kingdom; 5 MRC Cancer Unit, University of Cambridge, Cambridge, United Kingdom; 6 MRC Biostatistics Unit, University of Cambridge, Cambridge, United Kingdom

**Keywords:** cancer, hyperthyroidism, hypothyroidism, Mendelian randomization, thyroid-stimulating hormone, thyroxine

## Abstract

Whether thyroid dysfunction plays a causal role in the development of cancer remains inconclusive. We conducted a two-sample Mendelian randomization study to investigate the associations between genetic predisposition to thyroid dysfunction and 22 site-specific cancers. Single-nucleotide polymorphisms associated with four traits of thyroid function were selected from a genome-wide association meta-analysis with up to 72,167 European-descent individuals. Summary-level data for breast cancer and 21 other cancers were extracted from the Breast Cancer Association Consortium (122,977 breast cancer cases and 105,974 controls) and UK Biobank (367,643 individuals). For breast cancer, a meta-analysis was performed using data from both sources. Genetically predicted thyroid dysfunction was associated with breast cancer, with similar patterns of associations in the Breast Cancer Association Consortium and UK Biobank. The combined odds ratios of breast cancer were 0.94 (0.91-0.98; *p* = 0.007) per genetically predicted one standard deviation increase in TSH levels, 0.96 (0.91-1.00; *p* = 0.053) for genetic predisposition to hypothyroidism, 1.04 (1.01-1.07; *p* = 0.005) for genetic predisposition to hyperthyroidism and 1.07 (1.02-1.12; *p* = 0.003) per genetically predicted one standard deviation increase in free thyroxine levels. Genetically predicted TSH levels and hypothyroidism were inversely with thyroid cancer; the odds ratios were 0.47 (0.30-0.73; *p* = 0.001) and 0.70 (0.51-0.98; *p* = 0.038), respectively. Our study provides evidence of a causal association between thyroid dysfunction and breast cancer (mainly ER-positive tumors) risk. The role of TSH and hypothyroidism for thyroid cancer and the associations between thyroid dysfunction and other cancers need further exploration.

## Introduction

Subclinical thyroid dysfunction is defined as abnormal serum thyroid-stimulating hormone (TSH) levels with physiologically normal free thyroxine levels in asymptomatic patients^1^ and is a common disorder among adults,^2^ particularly in older women.^2^ Considering the important role of thyroid hormones in cell proliferation and differentiation, thyroid dysfunction has been proposed as a potential and preventable risk factor for cancer, such as thyroid^3,4^ and breast cancer.^5,6^

Observational data on the associations between thyroid dysfunction and risk of cancer are conflicting. Low TSH levels were associated with an increased risk of thyroid carcinoma in one study,^3^ while it was stated that higher TSH levels were associated with a higher frequency and more advanced stage of thyroid cancer in another study.^7^ With regard to breast cancer, a meta-analysis of 13 case-control studies published until June 2016 found no association between hypothyroidism or hyperthyroidism and breast cancer risk.^8^ However, results from a nationwide population-based cohort study in Denmark showed that women diagnosed with hypothyroidism and hyperthyroidism had respectively lower and higher risk of breast cancer compared to the general population.^9^ Similarly, hyperthyroidism was associated with a significant increased risk of breast cancer mortality in a prospective cohort study of 75,076 US women.^10^ A limitation of available evidence is that observational findings are prone to be influenced by residual confounding and reverse causality, thereby impeding the causal inference on association between thyroid dysfunction and cancer risk.

Genetic variants explicitly associated with a potential risk factor (e.g., TSH levels) can be used as unbiased proxies for the risk factor to determine causality. This approach, named as Mendelian randomization (MR), is a genetic method that can strengthen the inference on the causal nature of exposureoutcome associations by diminishing the likelihood of confounding and eliminating reverse causality in conventional observational studies.^11^ This is because the genetic alleles associated with the exposure are randomly assorted at conception and thus unrelated to self-selected lifestyle and environmental factors and are not modified by disease. Given the controversy and uncertainty of the role of thyroid dysfunction for cancer, we conducted a two-sample Mendelian randomization study to explore the causal associations of four indicators of thyroid function and dysfunction, including circulating TSH levels, hypothyroidism, hyperthyroidism and free thyroxine levels, with overall cancer and 22 site-specific cancers.

## Materials and Methods

### Assumptions of MR study and study design overview

**Figure 1:**
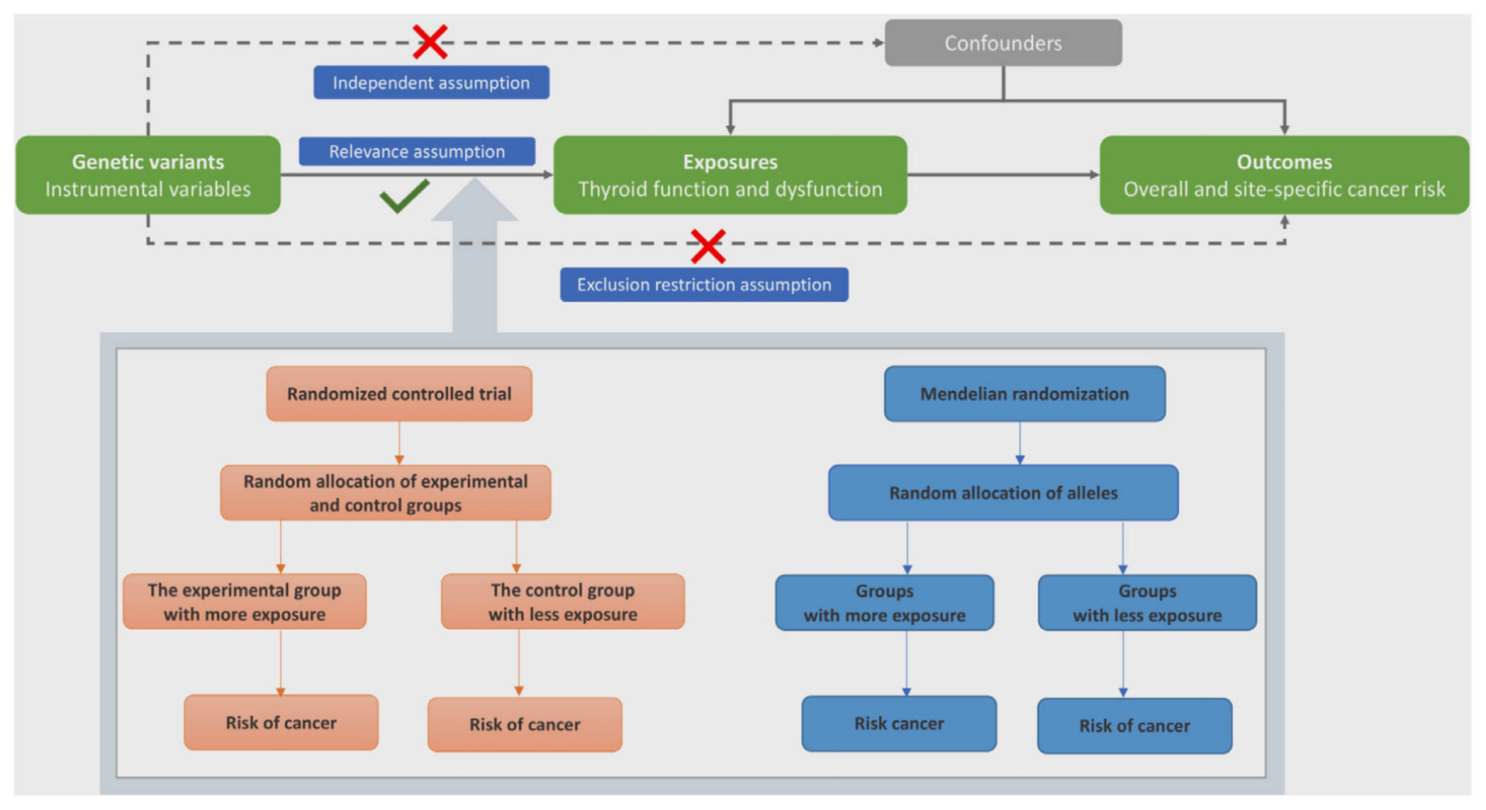
Assumptions of MR study and study design overview. [Color figure can be viewed at wileyonlinelibrary.com]

There are three key assumptions for MR analysis: (i) the genetic variants used as instrumental variables should be robustly associated with the risk factor of interest (Relevance assumption); (ii) the used genetic variants should not be associated with potential confounders (Independent assumption); and (iii) the genetic variants should affect the risk of the outcome only through the risk factor, not *via* alternative pathways (Exclusion restriction assumption). The present two-sample MR study was based on summary-level data from the ThyroidOmics Consortium,^12^ the Breast Cancer Association Consortium (BCAC)^13^ and the UK Biobank^14^ (Supporting Information Table S1). Assumptions of MR study and study design are shown in Figure 1. The original genome-wide association studies (GWASs) had been approved by corresponding ethical committee and all participants provided informed consent. The present study was approved by the Swedish Ethical Review Authority.

### Instrumental variable selection

Single-nucleotide polymorphisms (SNPs) associated with TSH (*n* = 61), hypothyroidism (*n* = 8), hyperthyroidism (*n* = 8) and free thyroxine levels (*n* = 31) at the genome-wide significance level (*p* < 5 × 10^−8^) were identified from a meta-analysis of GWASs of thyroid function and dysfunction with up to 72,167 individuals of European ancestry in both discovery and replication stages^12^ (Supporting Information Table S1). One TSH-related SNP located in the *ABO* locus was excluded due to pleiotropic effects (blood group is associated with many cancers,^15,16^ through effects independent of TSH), leaving 60 SNPs as instrumental variables for TSH levels. An SNP in the *GLIS1* gene showed genome-wide significant association with both TSH and free thyroxine, and SNPs in *FOXE1, PDE8B* and *PDE10A* locus were associated with both hyperthyroidism and hypothyroidism. In addition, the original GWAS verified the associations of TSH and free thyroxine level with hyperthyroidism and hypothyroidism using a genetic risk score.^12^ Proxy SNPs were chosen at *R* ^2^ > 0.9 among CEU population by searching in the dataset of Division of Cancer Epidemiology and Genetics, National Cancer Institute.^17^ We harmonized all instrumental variables for each trait so that the effect alleles reflected the allele associated with an increased probability or level of exposure. All used SNPs were uncorrelated (*R* ^2^ < 0.01) and details of the included SNPs are displayed in Supporting Information Table S2.

### Outcome data sources

Summary-level data for the associations of the thyroid-associated SNPs with breast cancer were obtained from BCAC, including 228,951 individuals of European ancestry (122,977 breast cancer cases and 105,974 controls)^13^ and UK Biobank^14^ with 13,666 breast cancer cases. The GWAS based on the BCAC used 1000 Genomes Project (Phase 3) reference panel in imputation stage and adjusted for genetic principal components and country. From UK Biobank, summary-level data for the SNP-cancer associations were also derived for overall cancer and additionally 21 site-specific cancers using logistic regression models adjusted for age, sex and 10 genetic principal components. The analyses of UK Biobank were based on 367,643 participants after exclusion of related individuals (third-degree relatives or closer), low call rate and excess heterozygosity (3 or more standard deviations from the mean). Follow-up was until March 31, 2017, or death, and in total 75,037 cancer cases were included.

### Statistical analysis

The Wald method was used to estimate the ratio between the SNP-outcome and SNP-exposure estimates for each SNP. The ratio estimates for every used SNPs for one trait were combined by using the fixed-effects or multiplicative randomeffects inverse-variance weighted meta-analysis method. The inverse-variance weighted method can provide the most precise estimates but could be influenced by invalid instrumental variables and pleiotropic effects. Thus, for overall cancer as well as associations reaching the conventional significance level (*p* < 0.05), we further conducted three sensitivity analyses based on the weighted median, MR-Egger and MR-pleiotropy residual sum and outlier (MR-PRESSO) methods to examine and correct for possible pleiotropy. The weighted median method gives accurate estimates if at least 50% of the instrumental variables are valid.^18^ The MR-Egger regression can detect and adjust for pleiotropy albeit with low power.^19^ The MR-PRESSO test can detect possible outliers and estimations obtained from the MR-PRESSO analysis are corrected for horizontal pleiotropy *via* outlier removal.^20^ To increase the power for the analysis of breast cancer, a meta-analysis with fixed effects was performed to combine the data from the BCAC and UK Biobank. Odds ratios (ORs) of cancer risk were scaled to one standard deviation (SD) increase in genetically predicted TSH and free thyroxine levels and one-unit increase in the log OR of hypothyroidism and hyperthyroidism in all analyses. All statistical analyses were two-sided and performed in Stata/SE 15.0 and R 3.6.0 software. We did not use *p* values strictly to define statistical significance but interpreted the results based on the patterns of associations across the thyroid-related traits and the strengths of the associations.^21^

## Results

Seven of the thyroid-associated SNPs were unavailable in the UK Biobank dataset. Proxy SNPs were found for four SNPs, resulting in 58 SNPs in the analyses of TSH, 7 SNPs in the analyses of hypothyroidism, 8 SNPs in the analyses of hyperthyroidism and 31 SNPs in the analyses of free thyroxine. One SNP for TSH was not available in the dataset of the BCAC and no suitable proxy was found.

**Figure 2:**
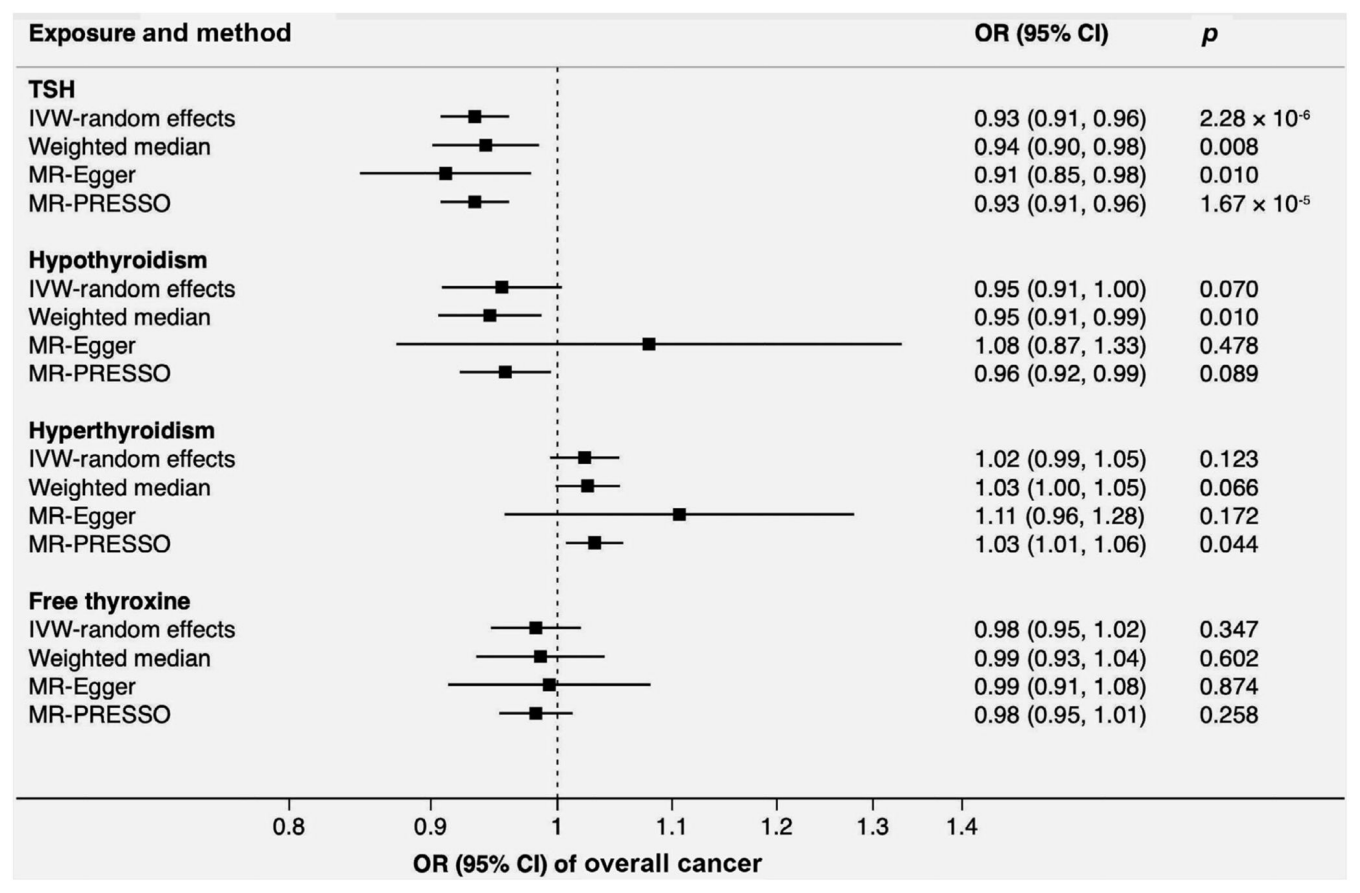
Associations of genetically predicted TSH and free thyroxine levels, hypothyroidism and hyperthyroidism with overall cancer in UK Biobank with 75,037 cancer cases at any site. Heterogeneity was observed in the analysis of hypothyroidism and hyperthyroidism. There was no detected pleiotropy in all MR-Egger analyses. Two and one outliers were detected and corrected in the MR-PRESSO analysis of hypothyroidism and hyperthyroidism, respectively. No outlier was detected in the analysis of TSH and free thyroxine. Abbreviations: CI, confidence interval; IVW, inverse-variance weighted; MR-PRESSO, Mendelian randomization-pleiotropy residual sum and outlier; OR, odds ratio; TSH, thyroid-stimulating hormone.

Genetically predicted TSH levels showed a consistent association with overall cancer across the different MR methods (Fig. 2). The OR was 0.93 (95% confidence interval [CI], 0.91-0.96; *p* = 2.28 × 10^−6^) per one standard deviation increase in TSH levels in the inverse variance weighted analysis. Results for genetic liability to hypothyroidism and hyperthyroidism were directionally consistent with those for TSH levels but less precise; in particular, the results from the MR-Egger analyses were very imprecise, indicative of very low power of this method.

**Figure 3:**
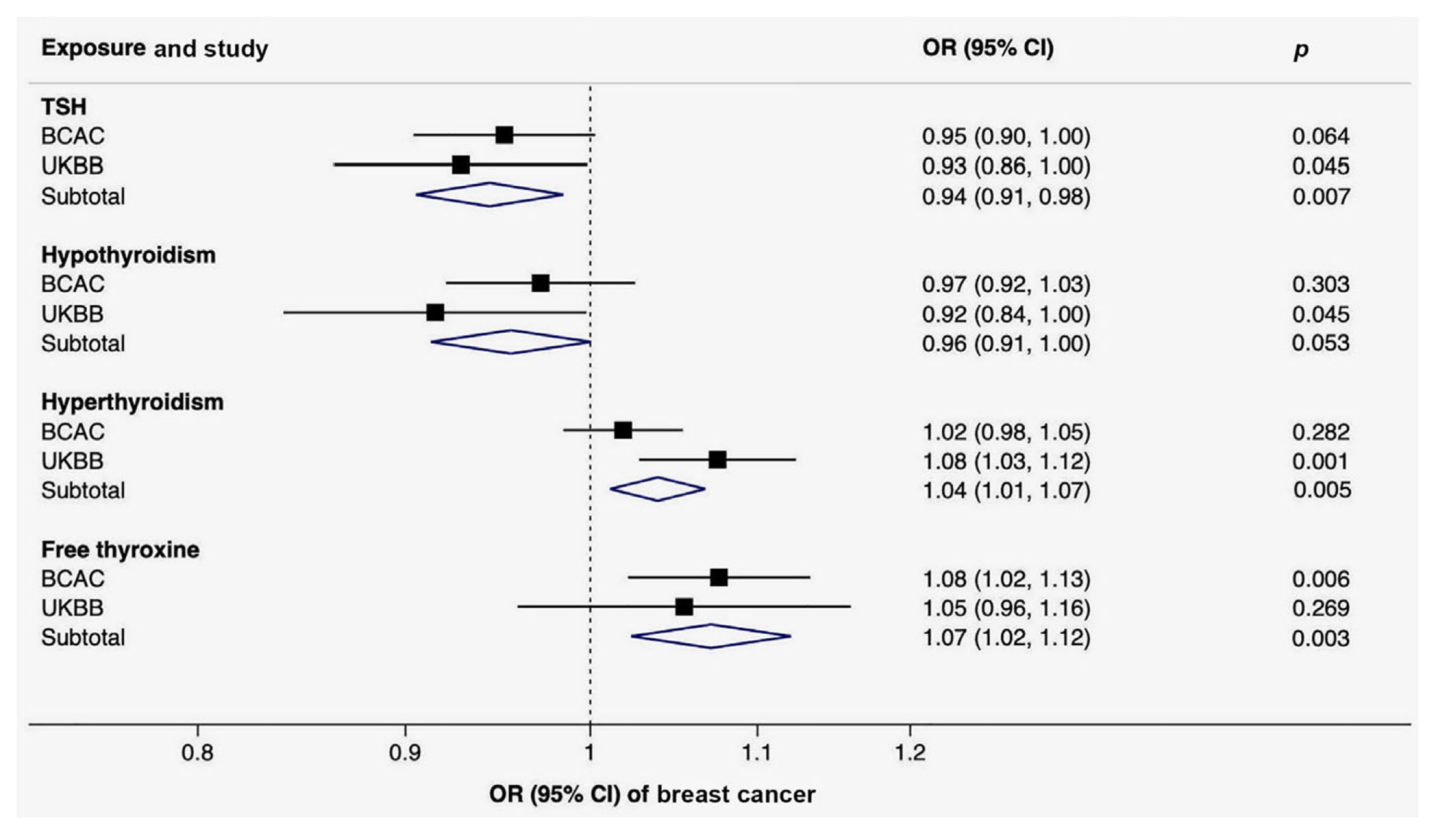
Meta-analysis of the associations of genetically predicted TSH and free thyroxine levels, hypothyroidism and hyperthyroidism with breast cancer. Abbreviations: BCAC, Breast Cancer Association Consortium; CI, confidence interval; TSH, thyroid-stimulating hormone; OR, odds ratio; UKBB, UK Biobank. [Color figure can be viewed at wileyonlinelibrary.com]

Genetically predicted TSH and free thyroxine levels as well as genetic predisposition to thyroid dysfunction were associated with breast cancer, with similar patterns of associations in the BCAC and UK Biobank (Table 1 and Fig. 3). In the meta-analysis combining the results from the BCAC and UK Biobank (136,643 breast cancer cases and 459,951 noncases), the combined ORs of breast cancer were 0.94 (95% CI 0.91-0.98; *p* = 0.007) per genetically predicted one SD increase in TSH levels, 0.96 (95% CI, 0.91-1.00; *p* = 0.053) per one-unit increase in log odds of hypothyroidism, 1.04 (95% CI, 1.01-1.07; *p* = 0.005) per one-unit increase in log odds of hyperthyroidism and 1.07 (95% CI, 1.02-1.12; *p* = 0.003) per genetically predicted one SD increase in free thyroxine levels (Fig. 3). The OR estimates were similar but less precise in the sensitivity analyses (Supporting Information Fig. S1). There was suggestive evidence that thyroid dysfunction, in particular hyperthyroidism and free thyroxine levels, was associated with estrogen-receptor (ER) positive but not negative tumors (Table 1).

**Figure 4:**
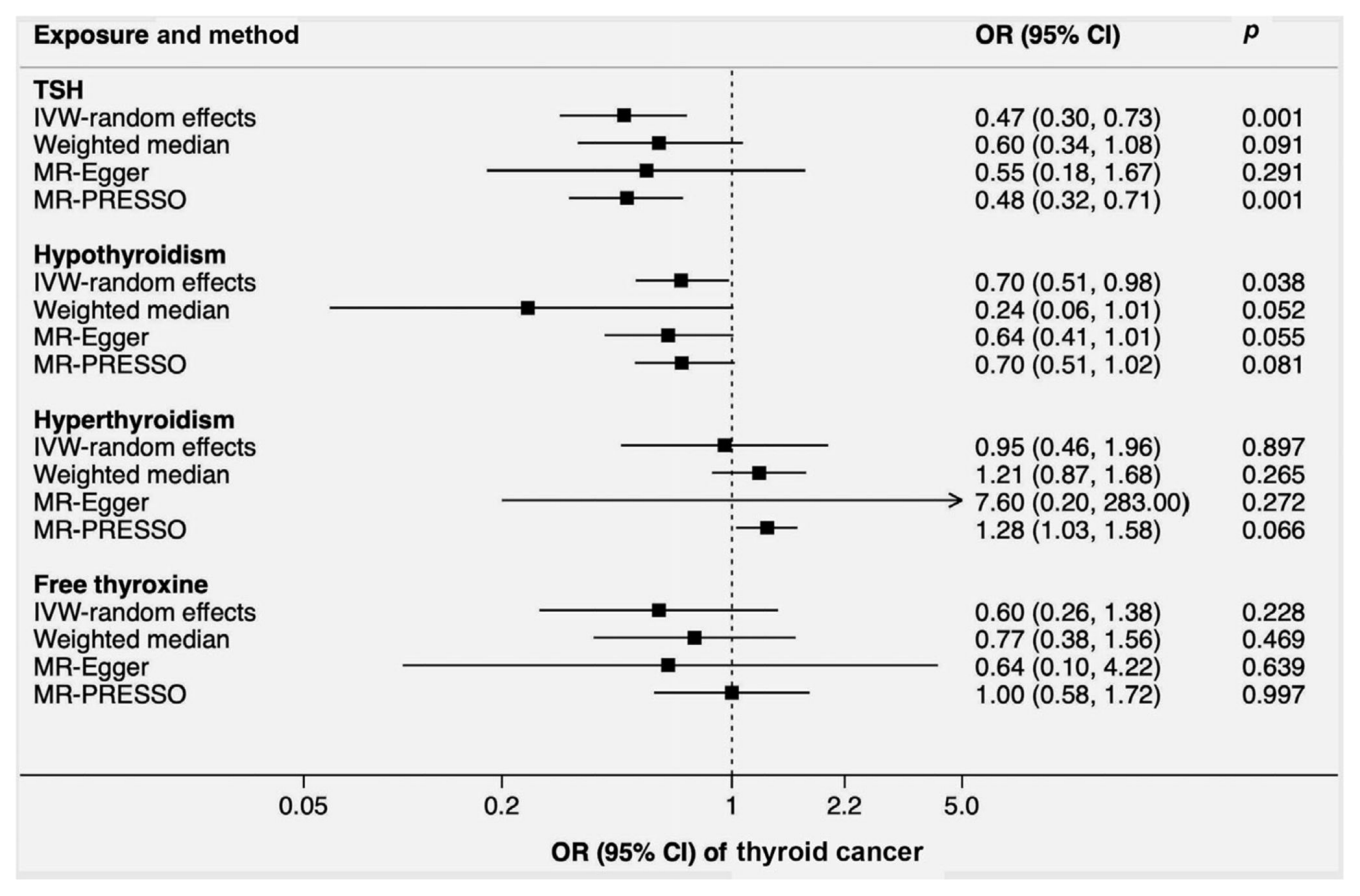
Associations of genetically predicted TSH and free thyroxine levels, hypothyroidism and hyperthyroidism with thyroid cancer. Heterogeneity was observed in the analysis of TSH, hyperthyroidism and free thyroxine. There was no detected pleiotropy in all MR-Egger analyses. Two, one and two outliers were detected and corrected in the MR-PRESSO analysis of TSH, hyperthyroidism and thyroxine, respectively. Abbreviations: CI, confidence interval; IVW, inverse-variance weighted; MR-PRESSO, Mendelian randomization-pleiotropy residual sum and outlier; OR, odds ratio; TSH, thyroid-stimulating hormone.

Genetically higher TSH levels and liability to hypothyroidism were associated with lower odds of thyroid cancer (Table 1 and Fig. 4). For one SD increase of TSH levels and one-unit increment of the log odds of hypothyroidism, the ORs of thyroid cancer were 0.47 (0.30-0.73; *p* = 0.001) and 0.70 (0.51-0.98; *p* = 0.038), respectively. Significant heterogeneity (*I* ^2^ = 39; *p* = 0.002) among estimates of individual SNPs was detected in the analysis of TSH. After removal of two outliers, the magnitude and the significance of the association between TSH and thyroid cancer remained in the MR-PRESSO analysis (OR = 0.48, 0.32-0.77; *p* = 0.001; Fig. 4). The associations were similar in sensitivity analyses using the weighted median and MR-Egger methods (Fig. 4).

There was no clear pattern of associations of genetically predicted thyroid dysfunction with the other 20 cancers studied (Table 1). Nevertheless, there was suggestive evidence of inverse associations of genetically predicted TSH levels with uterine and prostate cancer; an inverse association between genetic liability to hyperthyroidism and brain cancer; and inverse associations between free thyroxine levels and ovarian and bladder cancer and melanoma.

## Discussion

In the present study, we found evidence of a causal inverse association between TSH levels and overall cancer. Furthermore, increased TSH levels and hypothyroidism were associated with a decreased odds of breast cancer (mainly ER-positive tumors) and thyroid cancer, whereas hyperthyroidism and increased free thyroxine levels were associated with a higher odds of breast cancer (mainly ER-positive tumors). We found limited evidence supporting causal associations of thyroid dysfunction with 20 other cancers.

Observational studies have found that thyroid disorder and thyroid hormone levels were related to risk of overall cancer.^10,22–24^ Consistent with our findings, a 9-year cohort study of 29,691 individuals without previously known thyroid disease found that participants with low TSH levels had increased cancer risk compared to the euthyroid reference group after adjustment of age, sex and smoking status.^24^ Another cohort study of 75,076 US women and 30-year follow-up period showed that women with hyperthyroidism had an elevated risk of cancer, especially breast and ovarian cancer.^10^ In contrast, a cohort study of 115,746 Asians found that subclinical hypothyroidism was associated with increased cancer mortality over a 10-year follow-up period.^25^

In line with our MR results, most observational prospective studies have found that hypothyroidism is associated with a lower risk of breast cancer,^9,26^ whereas hyperthyroidism^9,10^ and high free thyroxine levels^5,27,28^ are associated with an increased breast cancer risk, especially among overweight^27^ and postmenopausal women.^28^ TSH levels were inversely associated with breast cancer risk in one study^28^ but not in two other.^5,27^ On the contrary, two meta-analyses based on data from 12 or 13 case-control studies showed no association of hypothyroidism or hyperthyroidism with odds of breast cancer.^8,29^ The inconsistency among studies may be attributable to reverse causation bias in the case-control studies, measurement bias (causing dilution of the effect), or inadequate power. In this MR study, results for thyroid function in relation to breast cancer were in the same direction in the BCAC and UK Biobank albeit less precise in UK Biobank. When combining the results from the two data sources, thereby increasing the sample size, all associations became statistically significant. In the present study, based on data from the BCAC, the associations of thyroid function and dysfunction with breast cancer risk were mainly observed for ER-positive tumors, though a suggestive association was also observed between hypothyroidism and ER-negative tumors. Considering that ER-positive tumors make up around 70% of total breast cancers in all populations, the observed association with overall breast cancer may reflect the association between thyroid dysfunction and ER-positive breast cancer. However, as data for ER status of the breast tumors were not available in UK Biobank, this difference could not be replicated in an independent population and needs confirmation.

The results of the present study are in line with most findings supporting a protective role of high TSH levels in thyroid cancer. An individual-matched nested case-control study with 1,482 individuals found an inverse association between physiologically high TSH levels and thyroid cancer.^30^ Similarly, another small case-control study showed that low levels of TSH might predispose to thyroid cancer.^3^ The possible mechanism behind the association may be TSH-specific mediated effect on thyroid tissue through cellular proliferation.^31^ However, a retrospective study including 3,791 patients with thyroidectomy found that increased serum TSH levels were related to higher odds of papillary thyroid cancer.^32^ Among patients with thyroid nodule, several studies have shown that high serum TSH levels increase thyroid cancer risk.^33,34^ In addition, TSH suppression therapy has been suggested as an efficient treatment for patients with high-risk thyroid cancer or recurrent tumor. However, in the present study, we observed an inverse association between higher TSH levels and thyroid cancer risk, which is in line with previous population-based epidemiological and genetic studies.^35,36^ Discrepancy may be explained by the differences in response to TSH in normal and cancerous tissues. A nationwide cohort study found no association of hypothyroidism with thyroid cancer among 63,143 patients with hypothyroidism compared to the general population but observed that benign thyroid disease was associated with higher standardized incidence ratio of thyroid cancer.^37^ A possible reason for the null association between hypothyroidism and thyroid cancer risk may be that once diagnosed with hypothyroidism, most patients are treated for the disorder and no longer hypothyroid, leading to altered cancer risk. This MR study found a possible inverse association between genetic liability to hypothyroidism and thyroid cancer, which needs verification due to a small number of thyroid cancer cases. In addition, even though there was no causal association between hyperthyroidism and thyroid cancer in our study, a systematic review documented that pathological hyperthyroid caused by Graves’ disease was associated with an increased risk of thyroid cancer.^38^

Some observational studies have reported associations of thyroid-related traits with other cancers, such as colorectal, prostate and lung cancer. Elevated TSH levels, hypothyroidism and decreased free thyroxine levels have been reported to associate with a higher risk of prostate cancer.^39,40^ A large-scale nested case-control study showed that hyperthyroidism and untreated hypothyroidism were associated with a modestly elevated risk of colorectal cancer.^41^ However, results for lung cancer have been conflicting with a positive^5^ or null^39^ association found between free thyroxine levels and lung cancer risk. The present MR study showed little evidence in support for an association of thyroid dysfunction with colorectal, prostate and lung cancers, except for an inverse association between TSH levels and prostate cancer. Possible explanations behind the discrepancy in results across studies may be residual confounding or reverse causality in the observational studies or an inadequate power in the present MR study owing to a small number of cases for these site-specific cancers.

Thyroid hormones are involved in physiological processes vital to normal metabolism, development and growth, and hypothyroidism is a known cause of growth retardation. Hence, not unexpectedly, genetic risk scores for elevated TSH and decreased free thyroxine levels are associated with decreased body height.^12^ Adult height is positively associated with risk of breast^42^ and thyroid cancer.^43^ This suggests that height (through more cells) might mediate the associations of thyroid dysfunction with breast and thyroid cancer, or that growth processes related to thyroid hormones are driving the positive association between height and cancer risk. Nevertheless, we did not detect any association of the thyroid hormones with other site-specific cancers that are also associated with height.^44,45^ Hence, it is reasonable to conclude that height is not biasing the results through vertical pleiotropy but that other dominating mechanisms explain the associations of thyroid dysfunction with breast and thyroid cancer. Previous studies indicated that body mass index might act as a mediator in the pathway from thyroid dysfunction to breast cancer.^46^ Nevertheless, genetically predicted TSH and free thyroxine levels were not associated with BMI using the genetic risk score analysis^12^ and we detected no such association of the liability to hypothyroidism and hyperthyroidism with BMI either (data not shown).

The exact mechanisms linking thyroid dysfunction to breast and thyroid cancer have not been clarified. There are several potential hypotheses, such as uptake and oxidation of iodine^6^ and a proliferative effect of triiodothyronine.^9^ Moreover, it has been shown that thyroxine is a proliferative factor *in vitro* for breast cancer cells and that thyroxine can promote nuclear estrogen receptor alpha-dependent proliferation of breast cancer cells bearing this receptor.^47^ Genetic studies have established a link connecting circulating TSH levels and thyroid cancer and found that *DIRC3, MBIP* and *NRG1* (encoding the signaling protein neuregulin 1) genes may play vital roles in this association.^36^ More studies focusing on the downstream of certain gene regions, such as key enzymes, metabolites and signal, transport and receptor proteins, are warranted for prevention strategy and therapy development.

This is the first MR study comprehensively assessing the associations of four thyroid function indicators with overall and 22 site-specific cancers. The major advantage of our study is the two-sample MR study design, which diminishes unobserved confounding and reverse causality potentially distorting the results of observational studies. The results were less likely to be biased by population stratification since we only used data from European populations, but this confined the transferability of our findings to other populations.

A major limitation of our study is that the number of cases was few for several cancers, leading to low precision of the estimates. Even though our results showed limited evidence supporting a causal association between thyroid dysfunction and site-specific cancers except breast and thyroid cancer, we cannot exclude that we may have missed weak associations owing to few cases. However, the precision was high in the analysis of breast cancer by combining the results from the BCAC and UK Biobank. Furthermore, the thyroid-related traits showed similar patterns of associations in both BCAC and UK Biobank, indicating that a false-positive finding is unlikely. The SNPs used as instrumental variables for the thyroid-function related traits have been reported to be associated with other factors, such as height, age at menarche, obesity-related traits, serum lipids, blood metabolite levels and serum urate.^12^ However, considering the biological roles of thyroid hormones, these factors are more likely to act as mediators (known as the vertical pleiotropy) in the pathway from thyroid function to cancer, which will not bias our findings. In addition, the consistency in results across sensitivity analyses and no detectable directional pleiotropy suggest a negligible distortion of the results by potential horizontal pleiotropy. Nevertheless, several thyroid-function related SNPs were directly associated with thyroid cancer. Thus, we cannot rule out that there are other direct pathways causing this cancer and consequently decreases TSH levels.

In summary, the present two-sample MR study strengthened the evidence of causal associations of thyroid function and dysfunction with risk of overall cancer and breast cancer (mainly ER-positive tumors). The observed inverse associations of circulating TSH levels and hypothyroidism with thyroid cancer need verification in other MR studies with larger number of cases. Along with the benefits of thyroid dysfunction treatment on cancer survival^48^ and cardiovascular diseases,^49^ it is suggested that treatment of subclinical and diagnosed hyperthyroidism may be an efficient cancer prevention strategy.

## Supplementary Material

**Additional Supporting Information** may be found in the online version of this article.

Supplementary Information

## Figures and Tables

**Table 1:** Associations of genetically predicted TSH and free thyroxine levels, hypothyroidism and hyperthyroidism with 22 cancers in random effects inverse-variance weighted model

		TSH		Hypothyroidism		Hyperthyroidism		Free thyroxine	
Cancer site or cancer	Cases	OR (95% Cl)	*P*	OR (95% Cl)	*P*	OR (95% Cl)	*P*	OR (95% Cl)	*P*
Sex-specific									
Breast (BCAC)	122,977	0.95 (0.90, 1.00)	0.064	0.97 (0.92, 1.03)	0.303	1.03 (1.00, 1.06)	0.053	1.07 (1.02, 1.13)	0.006
Breast ER+ (BCAC)	69,501	0.95 (0.90, 1.00)	0.066	0.98 (0.93, 1.04)	0.553	1.04 (1.00, 1.07)	0.026	1.08 (1.02, 1.14)	0.005
Breast ER− (BCAC)	21,468	1.02 (0.95, 1.09)	0.585	0.95 (0.91, 1.00)	0.067	0.99 (0.96, 1.03)	0.670	1.04 (0.96, 1.13)	0.297
Breast (ĲKBB)	13,666	0.93 (0.86, 1.00)	0.045	0.92 (0.84, 1.00)	0.045	1.08 (1.03, 1.12)	0.001	1.05 (0.96, 1.16)	0.269
Uterus	1,931	0.83 (0.70, 0.98)	0.025	0.93 (0.80, 1.08)	0.331	1.10 (0.96, 1.26)	0.177	1.06 (0.85, 1.34)	0.591
Cervix	1,928	1.00 (0.84, 1.17)	0.953	0.94 (0.81, 1.09)	0.432	1.09 (0.97, 1.22)	0.156	1.17 (0.93, 1.48)	0.169
Ovary	1,520	1.05 (0.89, 1.25)	0.552	0.96 (0.82, 1.14)	0.650	1.07 (0.91, 1.25)	0.408	0.79 (0.63, 1.00)	0.049
Prostate	7,872	0.91 (0.84, 0.99)	0.026	0.96 (0.89, 1.04)	0.305	0.99 (0.93, 1.06)	0.820	1.06 (0.93, 1.20)	0.400
Testis	735	0.89 (0.69, 1.16)	0.388	0.92 (0.71, 1.19)	0.520	1.01 (0.85, 1.20)	0.883	0.97 (0.70, 1.35)	0.859
Gastrointestinal tract									
Esophagus	843	0.80 (0.64, 1.01)	0.061	0.91 (0.72, 1.14)	0.393	1.08 (0.89, 1.31)	0.463	1.28 (0.94, 1.75)	0.122
Stomach	736	0.97 (0.76, 1.23)	0.788	0.91 (0.72, 1.15)	0.440	1.06 (0.89, 1.25)	0.529	1.12 (0.81, 1.56)	0.487
CoIorectum	5,486	1.00 (0.91, 1.09)	0.915	0.94 (0.80, 1.10)	0.454	0.97 (0.91, 1.04)	0.390	1.02 (0.89, 1.18)	0.743
Pancreas	1,264	0.92 (0.76, 1.11)	0.366	1.04 (0.86, 1.24)	0.698	1.10 (0.96, 1.25)	0.166	0.99 (0.77, 1.28)	0.959
Liver	324	0.82 (0.55, 1.23)	0.336	0.76 (0.45, 1.30)	0.322	1.14 (0.78, 1.65)	0.496	0.82 (0.46, 1.46)	0.509
Biliary tract	387	1.18 (0.84, 1.66)	0.329	1.04 (0.75, 1.44)	0.814	0.93 (0.73, 1.18)	0.552	1.33 (0.84, 2.08)	0.223
Urinary tract									
Bladder	2,588	1.14 (0.97, 1.33)	0.116	0.99 (0.87, 1.13)	0.929	0.93 (0.79, 1.09)	0.379	0.82 (0.68, 0.99)	0.036
Kidney	1,310	0.90 (0.74, 1.08)	0.257	0.91 (0.74, 1.13)	0.402	1.09 (0.95, 1.24)	0.212	0.97 (0.76, 1.25)	0.835
Blood/bone marrow/lymph									
Leukemia	1,403	0.90 (0.76, 1.08)	0.254	0.96 (0.81, 1.14)	0.639	1.01 (0.89, 1.14)	0.926	0.98 (0.78, 1.25)	0.921
Non-Hodgkin lymphoma	2,296	1.03 (0.90, 1.18)	0.687	0.87 (0.74, 1.01)	0.075	1.02 (0.93, 1.12)	0.663	0.88 (0.73, 1.06)	0.190
Multiple myeloma	656	0.99 (0.75, 1.30)	0.917	1.13 (0.88, 1.46)	0.328	0.95 (0.79, 1.14)	0.582	0.97 (0.68, 1.37)	0.853
Other									
Brain	810	0.92 (0.72, 1.17)	0.497	0.82 (0.61, 1.09)	0.168	1.24 (1.05, 1.46)	0.010	0.82 (0.59, 1.12)	0.207
Head and neck	1,615	0.95 (0.80, 1.12)	0.528	0.98 (0.76, 1.26)	0.878	0.95 (0.85, 1.07)	0.398	1.13 (0.89, 1.43)	0.308
Lung	2,838	1.00 (0.88, 1.14)	0.964	1.03 (0.91, 1.16)	0.648	1.07 (0.96, 1.18)	0.224	0.98 (0.79, 1.23)	0.880
Melanoma	4,869	1.03 (0.93, 1.14)	0.614	1.00 (0.88, 1.13)	0.997	1.04 (0.96, 1.12)	0.356	0.85 (0.73, 0.99)	0.040
Thyroid	375	0.47 (0.30, 0.73)	0.001	0.70 (0.51, 0.98)	0.038	0.95 (0.46, 1.96)	0.897	0.60 (0.26, 1.38)	0.228

Abbreviations: BCAC, Breast Cancer Association Consortium; CI, confidence interval; ER, estrogen receptor; OR, odds ratio; TSH, thyroid-stimulating hormone; UKBB, UK Biobank.

## Data Availability

Data for thyroid function can be obtained from the GWAS (accession code phs000930 in dbGaP, http://locuszoom.sph.umich.edu/genform.php).^12^ Summary-level data from BCAC^13^ are publicly available (http://bcac.ccge.medschl.cam.ac.uk/). UK Biobank^14^ data are available through application (https://www.ukbiobank.ac.uk/). Summary-level data for the used SNPs in the present study are available upon a reasonable request to the corresponding author.

## What’s new?

Whether thyroid dysfunction plays a causal role in the development of cancer remains inconclusive. This two-sample Mendelian randomization study investigated the associations between genetic predisposition to thyroid dysfunction and 22 site-specific cancers. Evidence of a causal inverse association was found between thyroid-stimulating hormone levels and overall cancer. Increased thyroid-stimulating hormone levels and hypothyroidism were associated with decreased risk of thyroid and breast (mainly ER-positive) cancer, whereas hyperthyroidism and increased free thyroxine levels were associated with a higher risk of breast (mainly ER-positive) cancer. The results suggest that treatment of subclinical and diagnosed hyperthyroidism may be an efficient cancer prevention strategy.

## Abbreviation

BCAC: Breast Cancer Association Consortium
CI: confidence interval
ER: estrogen-receptor
GWAS: genome-wide association study
MR: Mendelian randomization
MR-PRESSO: MR-pleiotropy residual sum and outlier
OR: odds ratio
SD: standard deviation
SNPs: single-nucleotide polymorphisms
TSH: thyroid-stimulating hormone
