# Supplementary material for: Causal associations of thyroid function and dysfunction with overall, breast and thyroid cancer: A two-sample Mendelian randomization study
Source: Int J Cancer. Author manuscript; Available in PMC 2021 Aug 24. (PMC7611568; doi:10.1002/ijc.32988)
Supplement: Supplementary Information [file EMS133103-supplement-Supplementary_Information.pdf]

Supplements

Causal associations of thyroid function and dysfunction with overall, breast and thyroid cancer: a two-sample Mendelian randomization study

Shuai Yuan, Siddhartha Kar, Mathew Vithayathil, Paul Carter, Amy M. Mason, Stephen Burgess, Susanna C. Larsson

Supplementary Table 1. Information of included studies and consortia
Supplementary Table 2. Detailed information of SNP used as instrumental variable for four thyroid-related traits

Supplementary Figure 1. Meta-analysis of the associations of genetically predicted TSH and free thyroxine levels, hypothyroidism and hyperthyroidism with breast cancer in sensitivity analyses based on the weighted median, MR-Egger, and MR-PRESSO methods

Supplementary Table 1. Information of included studies and consortia

Exposure/Outcome	Consortium or cohort study	Participants	Web source if publicly available
Thyroid function	The ThyroidOmics Consortium	72 167 European-descent individuals (22 independent cohorts with 54 288 individuals for the thyroid stimulating hormone analyses, and from 19 cohorts with 49 269 individuals for free thyroxine, 53 423 individuals (3440 cases) for hypothyroidism, and 51 823 individuals (1840 cases) for hyperthyroidism)	https://transfer.sysepi.medizin.uni-greifswald.de/thyroidomics/
Breast cancer	The Breast Cancer Association Consortium	228 951 European-descent individuals, including 122 977 all breast cancer cases (69 501 ER+ and 21 468 ER-) and 105 974 controls	http://bcac.ccge.medschl.cam.ac.uk/
Overall cancer and 22 site-specific cancers [a]	The UK Biobank	367 643 unrelated European-descent individuals (75 037 cancer cases and 292 606 controls)	https://www.ukbiobank.ac.uk/

[a] The numbers of cases for individual cancer in the UK Biobank are listed in the Manuscript Table 1.

Supplementary Table 2. Detailed information of SNP used as instrumental variable for four thyroid-related traits

Trait	SNP	Chr	Position	Nearby gene	EA	NEA	EAF	Beta [a]	SE	P	N	Proxy/Exclusion
TSH	rs12089835	1	19771438	*CAPZB*	T	C	0.35	0.073	0.007	1.3E-28	51018	
TSH	rs10917469	1	19843576	*CAPZB*	A	G	0.16	0.111	0.009	4.0E-39	51018	
TSH	rs74804879	1	19862320	*CAPZB*	T	C	0.35	0.050	0.007	1.2E-14	51018	
TSH	rs334725	1	61610049	*NFIA*	A	G	0.05	0.174	0.015	2.4E-32	51018	
TSH	rs17020122	1	108357391	*VAV3*	T	C	0.09	0.104	0.011	5.3E-20	51018	
TSH	rs16856540	2	217580413	*IGFBP5*	C	T	0.16	0.055	0.008	7.8E-11	52047	
TSH	rs13015993	2	217625523	*IGFBP5*	A	G	0.27	0.082	0.007	4.5E-32	52047	
TSH	rs1663070	3	12239852	*SYN2*	C	T	0.26	0.046	0.007	3.5E-11	54288	
TSH	rs6535624	4	149587905	*NR3C2*	A	G	0.44	0.042	0.006	1.6E-11	52047	
TSH	rs11732089	4	149665602	*NR3C2*	T	C	0.20	0.115	0.008	1.7E-51	52047	
TSH	rs62362610	5	76439961	*PDE8B*	C	G	0.08	0.073	0.012	7.7E-10	53074	
TSH	rs1119208	5	76488613	*PDE8B*	T	C	0.37	0.046	0.006	6.6E-13	53074	
TSH	rs139424329	5	76495539	*PDE8B*	G	A	0.01	0.200	0.032	5.1E-10	45981	
TSH	rs2127387	5	76532571	*PDE8B*	A	G	0.41	0.144	0.006	1.1E-117	53074	
TSH	rs7702192	5	76554807	*PDE8B*	A	C	0.47	0.070	0.006	2.6E-30	53074	
TSH	rs113974964	5	76652403	*PDE8B*	C	T	0.05	0.124	0.015	2.1E-17	53074	
TSH	rs139149784	5	76660193	*PDE8B*	A	G	0.03	0.156	0.029	5.0E-08	49018	
TSH	rs182873197	5	76773148	*PDE8B*	C	T	0.05	0.080	0.014	1.7E-08	53074	rs116164048
TSH	rs744103	6	43805362	*VEGFA/LOC100132354*	A	T	0.31	0.092	0.007	6.7E-41	54288	
TSH	rs9381266	6	43905037	*VEGFA/LOC100132354*	T	C	0.26	0.073	0.007	1.8E-25	54288	
TSH	rs9497965	6	148521292	*SASH1*	T	C	0.40	0.044	0.006	9.8E-13	54288	
TSH	rs73022105	6	165973757	*PDE10A*	T	C	0.05	0.105	0.016	1.2E-11	52424	
TSH	rs1079418	6	166047034	*PDE10A*	A	G	0.31	0.101	0.007	8.2E-53	52424	
TSH	rs2439301	8	32433013	*NRG1*	G	A	0.23	0.059	0.008	8.2E-15	54288	
TSH	rs10814915	9	4290544	*GLIS3*	T	C	0.44	0.042	0.006	5.1E-12	54288	
TSH	rs8176645	9	136149098	*ABO*	A	T	0.34	0.052	0.006	3.9E-16	54288	Excluded [b]
TSH	rs200574439	10	101283330	*NKX2-3*	C	A	0.42	0.047	0.006	3.7E-13	54288	NA
TSH	rs12284404	11	45228686	*PRDM11*	G	A	0.27	0.067	0.007	2.5E-22	54288	
TSH	rs398745	14	36536181	*MBIP*	C	A	0.41	0.052	0.006	4.0E-17	54288	

TSH	rs2254613	14	36713154	*MBIP*	G	T	0.45	0.035	0.006	3.4E-08	54288	
TSH	rs8015085	14	93585331	*ITPK1*	A	G	0.21	0.067	0.008	2.4E-18	54288	
TSH	rs17477923	15	49711185	*FAM227B/FGF7*	T	C	0.26	0.083	0.007	2.6E-33	54288	
TSH	rs11639111	15	49749735	*FAM227B/FGF7*	T	C	0.41	0.045	0.006	3.6E-13	54288	
TSH	rs13329353	15	89113877	*DET1*	T	C	0.32	0.061	0.007	5.2E-21	54288	
TSH	rs17767491	16	79745487	*MAF*	A	G	0.32	0.088	0.007	3.4E-42	54288	
TSH	rs1042673	17	70121339	*SOX9*	G	A	0.48	0.055	0.006	3.6E-19	54288	
TSH	rs963384	17	70369758	*SOX9*	T	C	0.46	0.035	0.006	2.8E-08	54288	
TSH	rs4804413	19	7222655	*INSR*	T	C	0.44	0.053	0.006	8.6E-18	51942	
TSH	rs1203944	20	22596879	*FOXA2*	C	T	0.23	0.051	0.007	2.4E-12	54288	
TSH	rs6724073	2	218236786	*DIRC3*	T	C	0.26	0.051	0.008	1.3E-10	52047	
TSH	rs28502438	3	149220109	*TM4SF4*	T	C	0.43	0.034	0.006	3.7E-08	54288	
TSH	rs13100823	3	185514088	*IGF2BP2*	C	T	0.31	0.041	0.007	6.8E-10	54288	
TSH	rs59381142	3	193916181	*HES1*	G	A	0.24	0.058	0.008	1.7E-14	52048	
TSH	rs1265091	6	31108129	*PSORS1C1*	T	C	0.20	0.057	0.009	3.2E-11	47651	
TSH	rs56009477	8	23356964	*SLC25A37*	A	G	0.16	0.052	0.008	3.7E-10	54288	
TSH	rs10957494	8	70365025	*SULF1*	G	A	0.31	0.040	0.007	1.1E-09	54288	
TSH	rs118039499	8	133771635	*TG*	A	C	0.02	0.184	0.024	2.0E-14	48736	
TSH	rs2739067	8	133951991	*TG*	G	A	0.40	0.042	0.006	2.4E-11	54288	
TSH	rs9298749	9	16214340	*C9orf92*	C	A	0.41	0.039	0.006	8.8E-10	54288	
TSH	rs11255790	10	8682180	*GATA3*	C	T	0.30	0.041	0.007	6.8E-10	54288	
TSH	rs4933466	10	89849519	*PTEN*	A	G	0.40	0.040	0.006	5.1E-10	54288	
TSH	rs4445669	11	115045237	*CADM1*	C	T	0.46	0.040	0.006	5.8E-11	54288	
TSH	rs7329958	13	24782080	*SPATA13*	C	T	0.35	0.044	0.007	1.1E-11	54288	
TSH	rs11159482	14	81490842	*TSHR*	T	C	0.09	0.085	0.013	6.3E-11	54288	
TSH	rs59334515	14	81594143	*TSHR*	C	T	0.22	0.054	0.007	1.1E-13	54288	
TSH	rs12893151	14	81619945	*TSHR*	C	A	0.22	0.062	0.008	1.0E-15	54288	
TSH	rs1045476	16	4015313	*ADCY9*	A	G	0.18	0.049	0.008	2.4E-09	54288	
TSH	rs30227	16	14405428	*MIR365A*	C	T	0.39	0.047	0.006	7.6E-14	54288	
TSH	rs77819282	17	44762589	*NSF*	A	G	0.24	0.045	0.007	1.1E-09	54288	rs199436
TSH	rs1157994	17	59338574	*BCAS3*	G	A	0.05	0.090	0.016	5.3E-09	50232	
TSH	rs12390237	X	3612081	*PRKX*	G	A	0.38	0.046	0.007	1.7E-11	36501	NA
Hypo	rs7032019	9	100548144	*FOXE1*	A	G	0.34	0.248	0.027	5.5E-20	53241	NA

Hypo	rs78495697	1	108355719	*VAV3*	T	C	0.09	0.316	0.041	2.6E-14	53241
Hypo	rs597808	12	111973358	*ATXN2/SH2B3*	A	G	0.49	0.180	0.026	3.5E-12	53241
Hypo	rs75491569	1	19836221	*CAPZB*	C	T	0.16	0.238	0.037	8.7E-11	53241
Hypo	rs11675342	2	1407628	*TPO*	T	C	0.43	0.160	0.025	1.5E-10	53241
Hypo	rs1382879	5	76521868	*PDE8B*	C	T	0.41	0.153	0.026	2.1E-09	48969
Hypo	rs2983514	6	166050119	*PDE10A*	A	G	0.33	0.154	0.027	1.6E-08	51313
Hypo	rs12449792	17	43302259	*FMNL1*	T	C	0.46	0.157	0.028	2.4E-08	46314
Hyper	rs2046045	5	76535811	*PDE8B*	T	G	0.40	0.329	0.034	4.4E-22	50420
Hyper	rs17477923	15	49711185	*FAM227B/FGF7*	C	T	0.28	0.245	0.034	6.1E-13	48508
Hyper	rs11038357	11	45237858	*PRDM11*	A	T	0.28	0.235	0.035	3.1E-11	48508
Hyper	rs8077245	17	70376177	*SOX9*	T	G	0.42	0.205	0.033	5.0E-10	48508
Hyper	rs2983514	6	166050119	*PDE10A*	G	A	0.35	0.199	0.032	5.2E-10	49745
Hyper	rs12138950	1	19839115	*CAPZB*	C	A	0.15	0.258	0.042	5.5E-10	51668
Hyper	rs925488	9	100546391	*FOXE1*	A	G	0.35	0.190	0.033	1.1E-08	48508
Hyper	rs66760320	6	43906255	*VEGFA/LOC100132354*	T	C	0.27	0.192	0.035	4.4E-08	51668
FT4	rs145019385	1	54252139	*DIO1*	T	C	0.02	0.181	0.032	1.1E-08	42463
FT4	rs12033572	1	54369674	*DIO1*	C	G	0.05	0.115	0.017	1.4E-11	49269
FT4	rs2235544	1	54375570	*DIO1*	A	C	0.48	0.139	0.007	4.2E-101	49269
FT4	rs954878	1	54578401	*DIO1*	G	A	0.45	0.058	0.007	4.8E-19	49269
FT4	rs6854291	4	170992760	*AADAT*	A	G	0.10	0.117	0.011	1.3E-24	47314
FT4	rs10739496	9	100552559	*FOXE1*	T	C	0.36	0.078	0.007	4.2E-30	49269
FT4	rs10984606	9	100739117	*FOXE1*	G	T	0.50	0.040	0.007	1.2E-09	49269
FT4	rs4842131	9	139092679	*LHX3*	C	T	0.45	0.104	0.008	7.7E-44	44811
FT4	rs55679545	9	139122363	*LHX3*	A	G	0.27	0.044	0.008	8.4E-09	49269
FT4	rs1080094	18	29173795	*SLC25A52*	G	A	0.40	0.042	0.007	4.1E-10	49269
FT4	rs113107469	18	29306737	*SLC25A52*	T	C	0.03	0.200	0.022	1.0E-19	49269
FT4	rs4954192	2	135632981	*ACMSD*	C	T	0.43	0.041	0.007	8.4E-09	44902
FT4	rs6785807	3	181718601	*SOX2-OT*	G	A	0.15	0.059	0.009	2.5E-10	49269
FT4	rs10946313	6	19381386	*ID4*	T	C	0.37	0.046	0.007	2.3E-11	49269
FT4	rs9356988	6	25777481	*SLC17A4*	G	A	0.27	0.051	0.007	3.6E-12	49269
FT4	rs137964359	6	26001742	*SLC17A4*	C	T	0.01	0.200	0.032	2.1E-10	49269
FT4	rs17185536	6	100620931	*LOC728012*	T	C	0.24	0.073	0.008	1.9E-19	49269
FT4	rs67583169	8	61212179	*CA8*	C	G	0.13	0.061	0.010	1.0E-10	49269

FT4	rs10119187	9	4223660	*GLIS3*	T	C	0.19	0.050	0.009	4.1E-09	49269	
FT4	rs10818937	9	127015440	*NEK6*	C	T	0.31	0.048	0.007	1.3E-11	49269	
FT4	rs11039355	11	47737501	*FNBP4*	C	T	0.34	0.039	0.007	3.5E-08	49269	rs11604825
FT4	rs4149056	12	21331549	*SLCO1B1*	C	T	0.16	0.051	0.009	1.3E-08	49269	
FT4	rs150816132	14	80464293	*DIO2*	G	A	0.01	0.220	0.040	3.5E-08	38640	
FT4	rs978055	14	80534869	*DIO2*	A	T	0.38	0.038	0.007	1.1E-08	49269	
FT4	rs225014	14	80669580	*DIO2*	T	C	0.36	0.054	0.007	1.8E-15	49269	
FT4	rs12323871	14	101852075	*DIO3OS*	C	T	0.18	0.047	0.008	1.4E-08	49269	
FT4	rs11626434	14	101998443	*DIO3OS*	C	G	0.36	0.058	0.007	4.1E-17	49269	
FT4	rs12907106	15	63873658	*USP3*	G	C	0.27	0.041	0.007	3.7E-08	49269	
FT4	rs8063103	16	12703395	*SNX29*	G	C	0.15	0.052	0.009	1.6E-08	49269	
FT4	rs11078333	17	16049626	*NCOR1*	A	T	0.47	0.051	0.007	9.9E-13	49269	
FT4	rs56069042	18	57914644	*MC4R*	A	G	0.04	0.106	0.019	1.2E-08	49269	

Chr indicates chromosome; EA, effect allele; EAF, effect allele frequency; FT4, free thyroxine; Hyper, hyperthyroidism; Hypo, hypothyroidism; NA, not available; NEA, non-effect allele; SE, standard error; SNP, single-nucleotide polymorphisms; TSH, thyroid-stimulating hormone.

[a] The beta coefficients represent the change in circulating levels of TSH (in SD, μm) and FT4 (in SD, μm) for each additional effect allele and in the log odds ratio of having hypothyroidism and hyperthyroidism.

[b] Excluded because the ABO (blood group) locus has multiple pleiotropic effects and is associated with several cancers through effects not mediated by TSH levels.

Supplementary Figure 1. Meta-analysis of the associations of genetically predicted TSH and free thyroxine levels, hypothyroidism and hyperthyroidism with breast cancer in sensitivity analyses based on the weighted median, MR-Egger, and MR-PRESSO methods

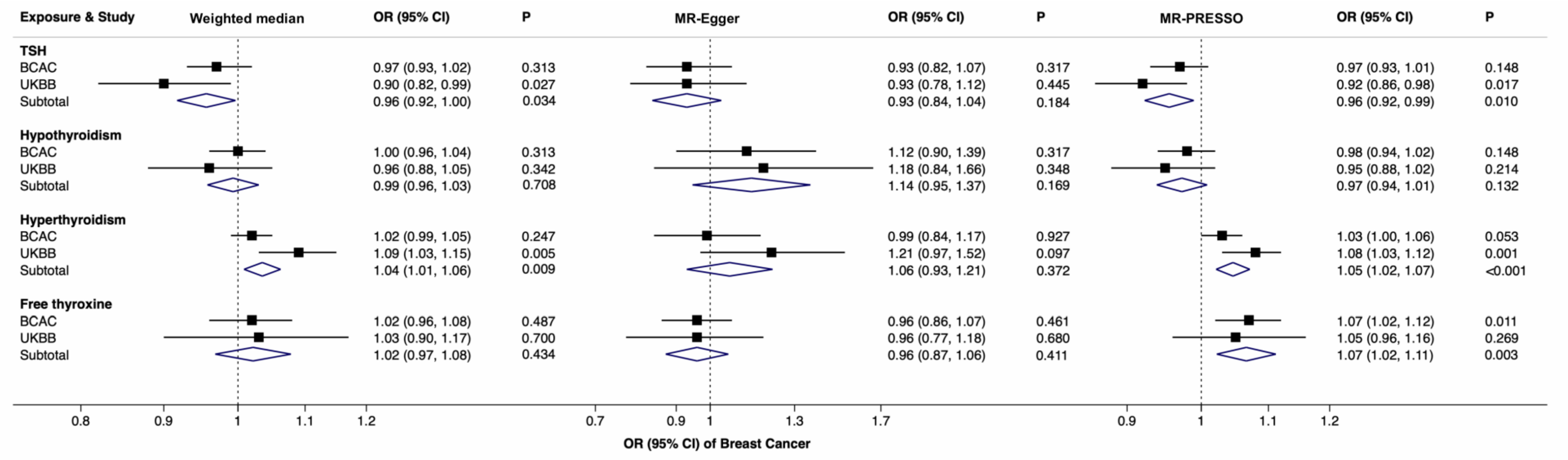

BCAC indicates Breast Cancer Association Consortium; CI; confidence interval; MR-PRESSO, Mendelian randomization-pleiotropy residual sum and outlier; TSH, thyroid-stimulating hormone; IVW, inverse-variance weighted; OR, odds ratio; SNP**,** single nucleotide polymorphism; UKBB, UK Biobank. Sensitivity analyses in BCAC: significant heterogeneity was detected in all analyses. However, pleiotropy was observed in the analysis of free thyroxine. Five, two and one outliers were detected and corrected for in the MR-PRESSO analysis of TSH, hypothyroidism and thyroxine, respectively. Sensitivity analyses in UKBB: significant heterogeneity was detected in the analysis of TSH and hypothyroidism. There was no observed pleiotropy. One outlier was detected and corrected in the MR-PRESSO analysis of TSH and hypothyroidism, respectively.
